# Supplementary material for: Shotgun Proteomics of Co-Cultured Leukemic and Bone Marrow Stromal Cells from Different Species as a Preliminary Approach to Detect Intercellular Protein Transfer
Source: Proteomes. 2023 Apr 5;11(2):15. doi: 10.3390/proteomes11020015 (PMC10123657; doi:10.3390/proteomes11020015)

Figure S1. Quality control charts and statistics for mass spectrometry data.

Quality control graphs for proteins identified in the CCRF-CEM cells

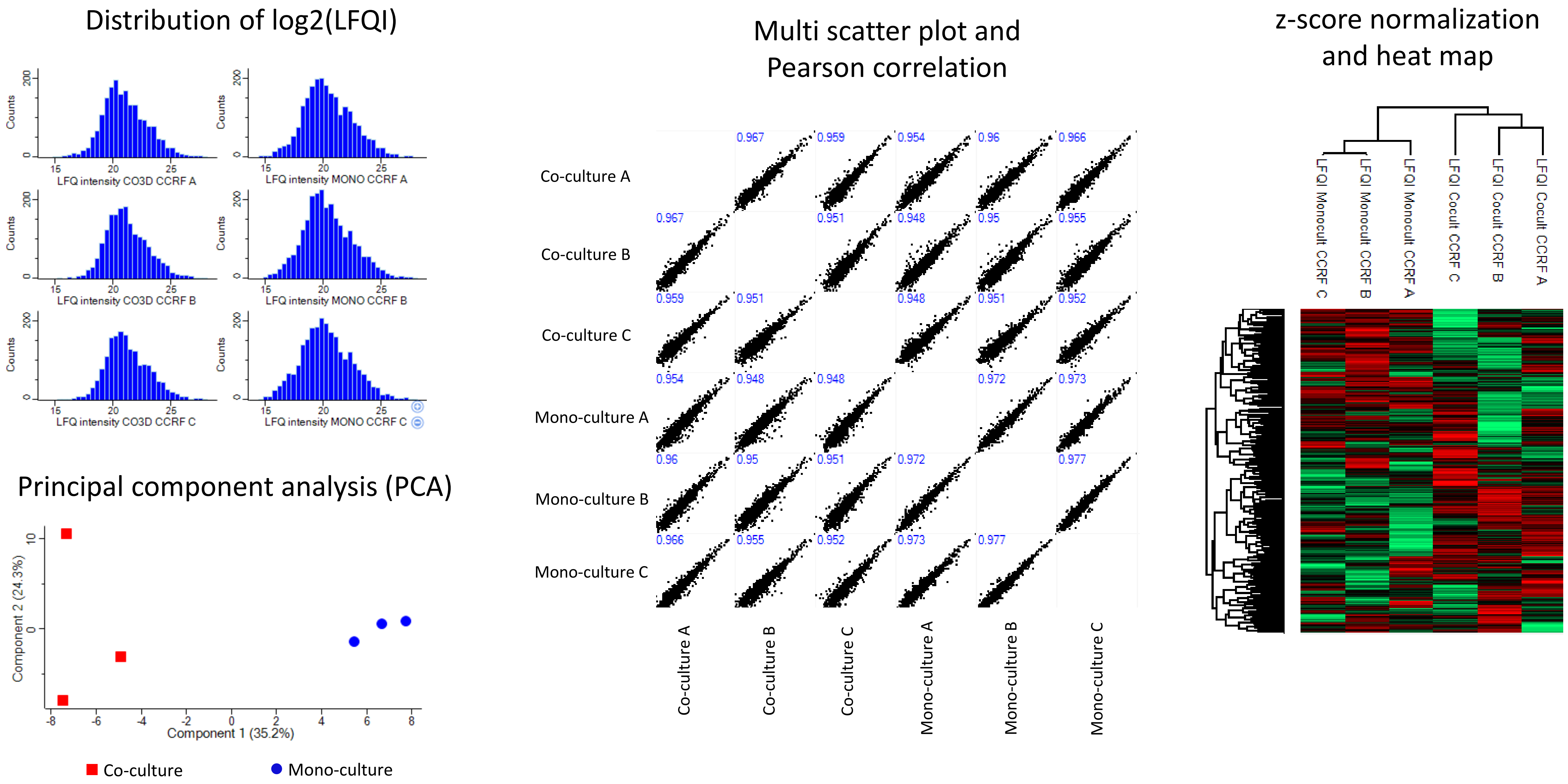

Quality control graphs for peptides identified in the CCRF-CEM cells

Distribution of log2(LFQI)

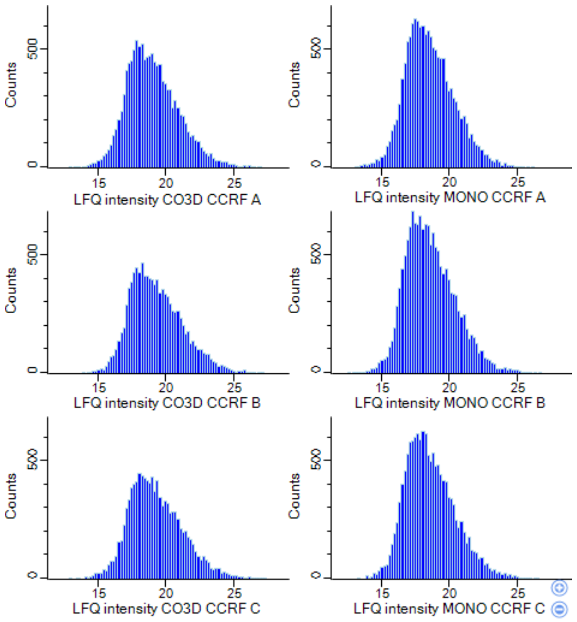

Principal component analysis (PCA)

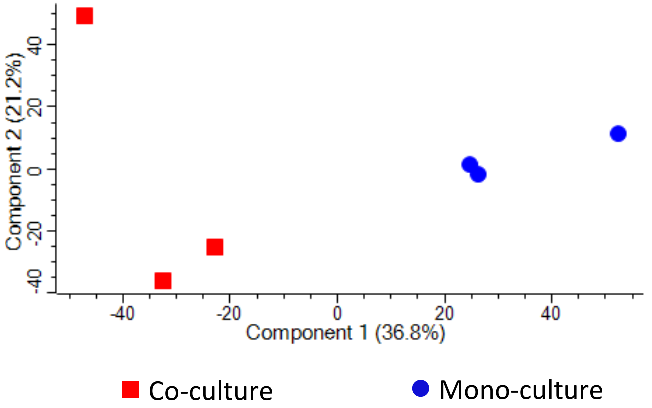

Multi scatter plot and Pearson correlation

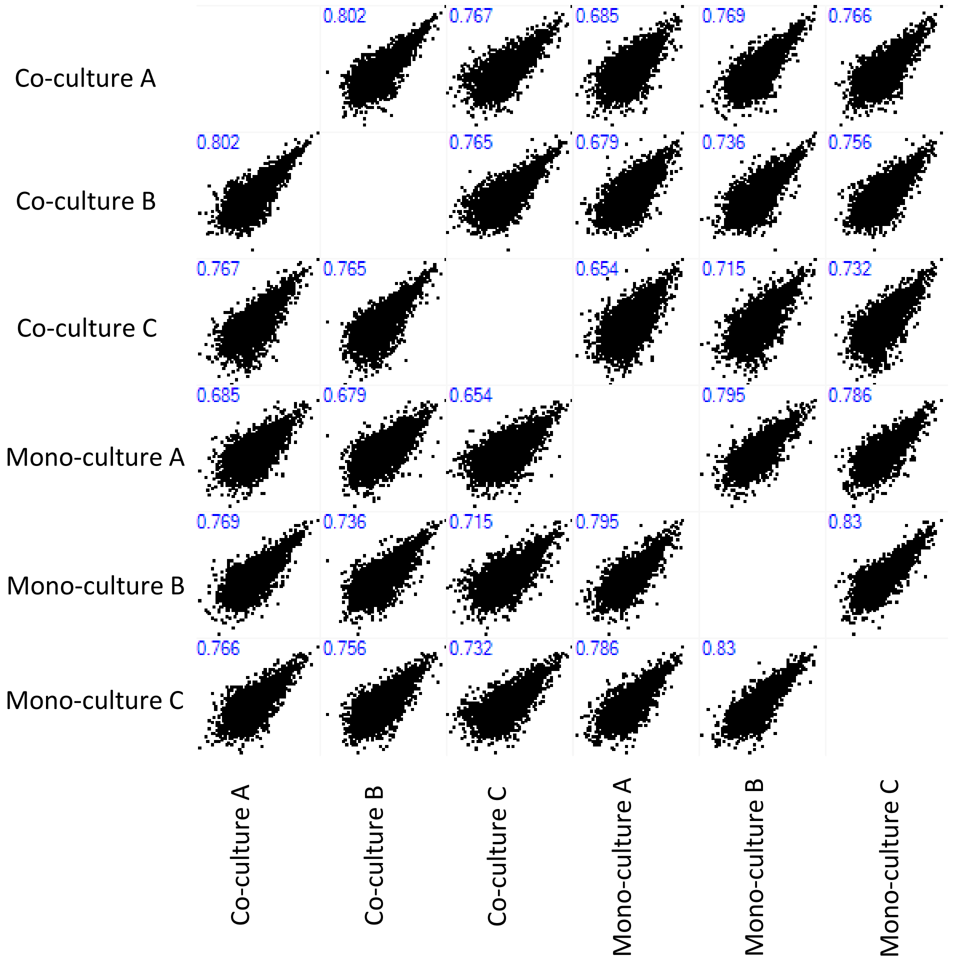

z-score normalization and heat map

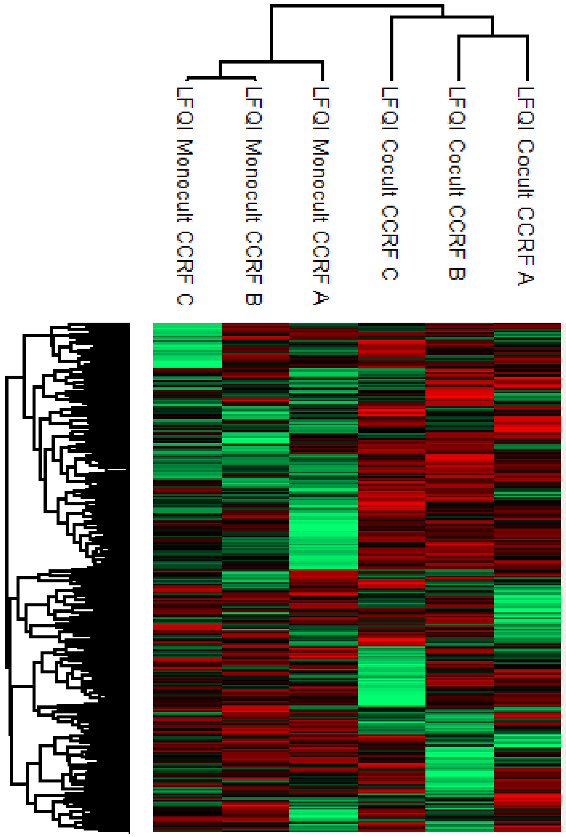

Quality control graphs for the 209 mouse peptides identified in human CCRF-CEM

Distribution of log2(LFQI)

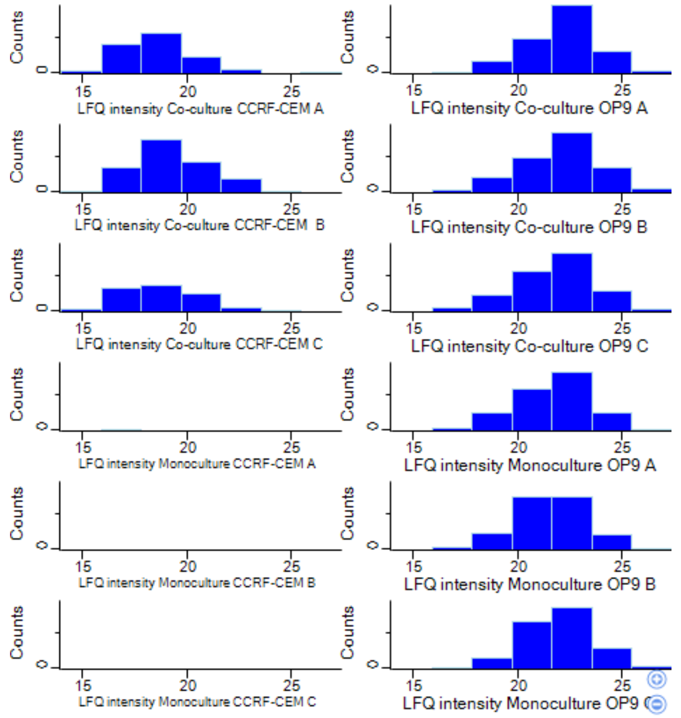

Multi scatter plot and Pearson correlation

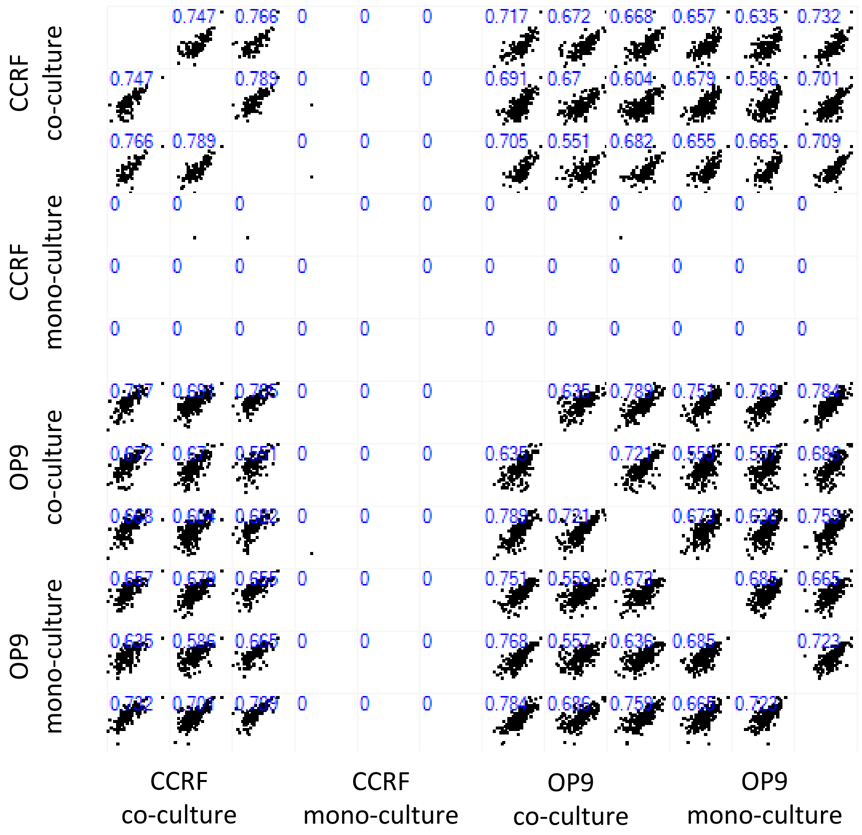

z-score normalization and heat map

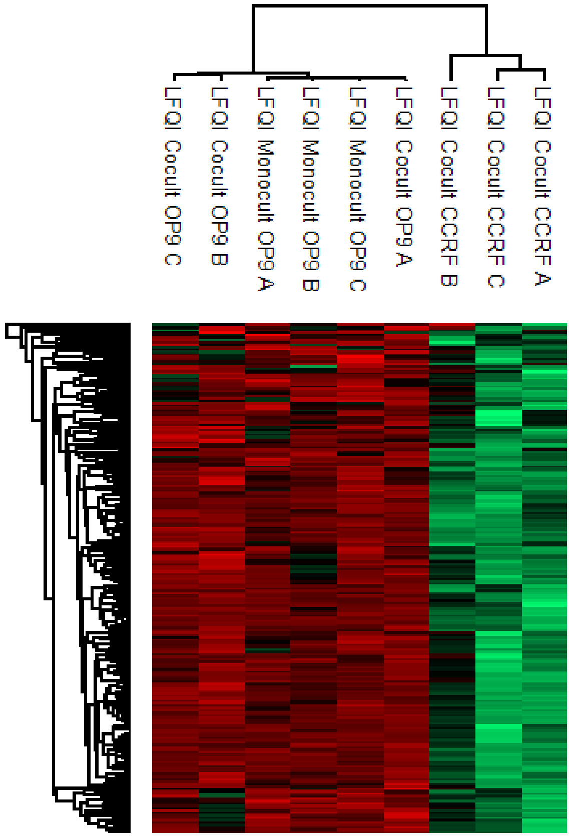

Principal component analysis (PCA)

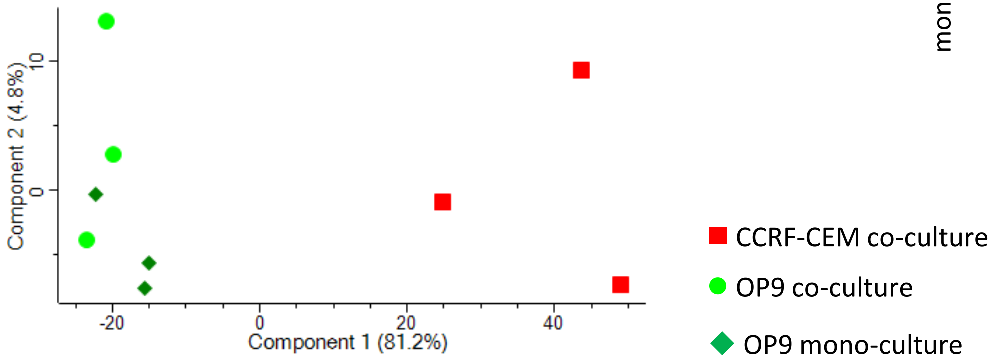

Plot of covariances of the label free quantification intensity values of proteins identified in the CCRF-CEM cells

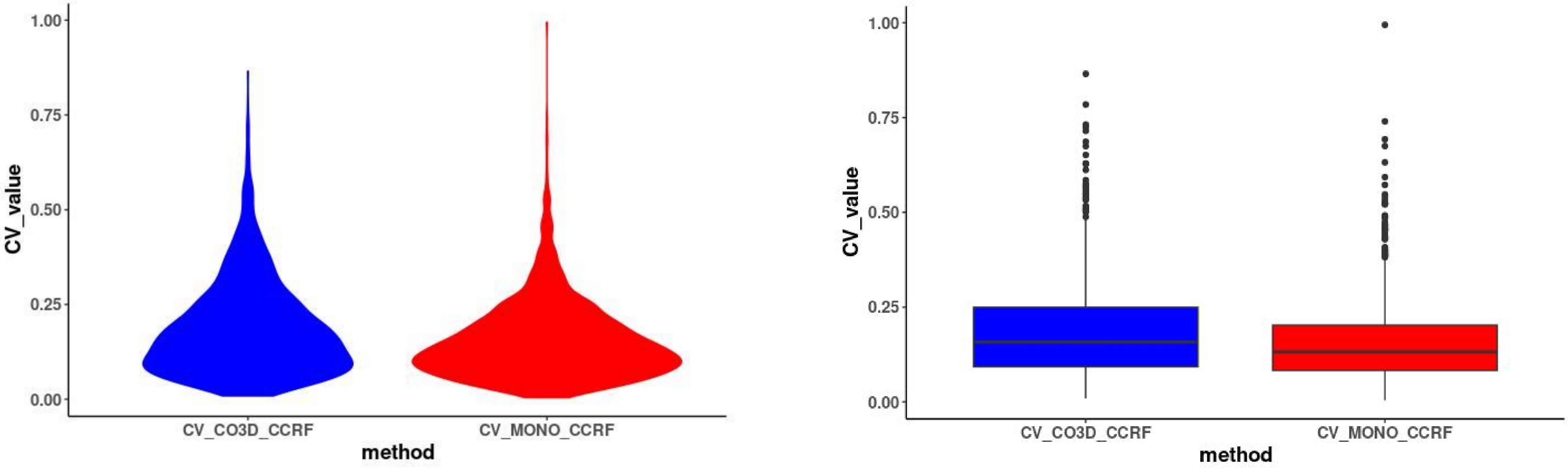

MS/MS spectra of selected mouse peptides detected in the suspension of co-cultued CCRF-CEM cells

In the sequences of mouse peptides, the residues in bold letters are different between the closest mouse and human peptides, and underlined residues were detected as y or b ions.

Sequence of mouse peptide	Sequence of the closest human peptide	UniProt ID	Gene name - mouse	Protein names
**I**LVNSI**TSLV**NTFVPSGK	**V**IVNSL**KNMI**NTFVPSGK	Q9WV54	Asah1	Acid ceramidase;Acid ceramidase subunit alpha;Acid ceramidase subunit beta

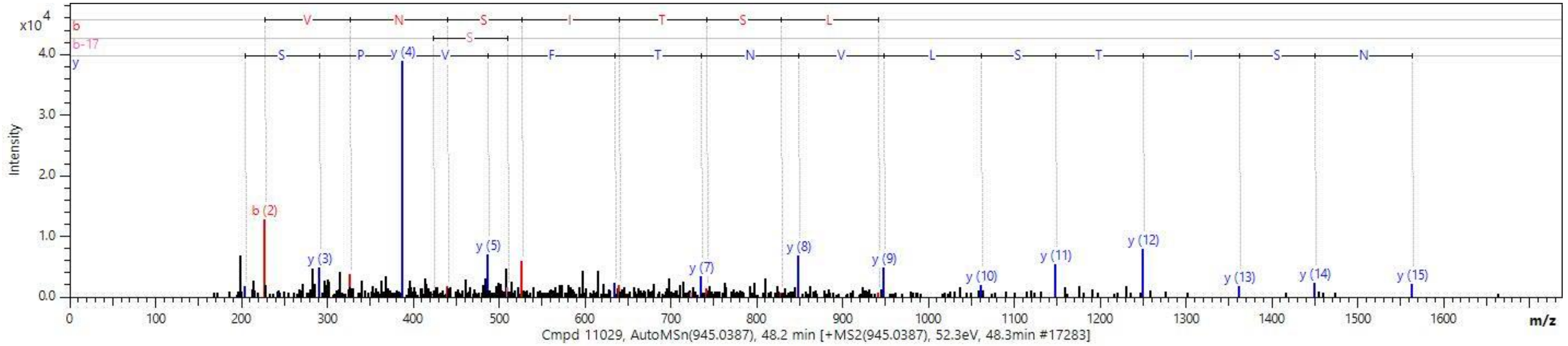

Sequence of mouse peptide	Sequence of the closest human peptide	UniProt ID	Gene name - mouse	Protein names
**VS**LAVL**N**PYI**K**	**FP**LAVL**K**PYI**H**	Q9DB20	Atp5o	ATP synthase subunit O, mitochondrial

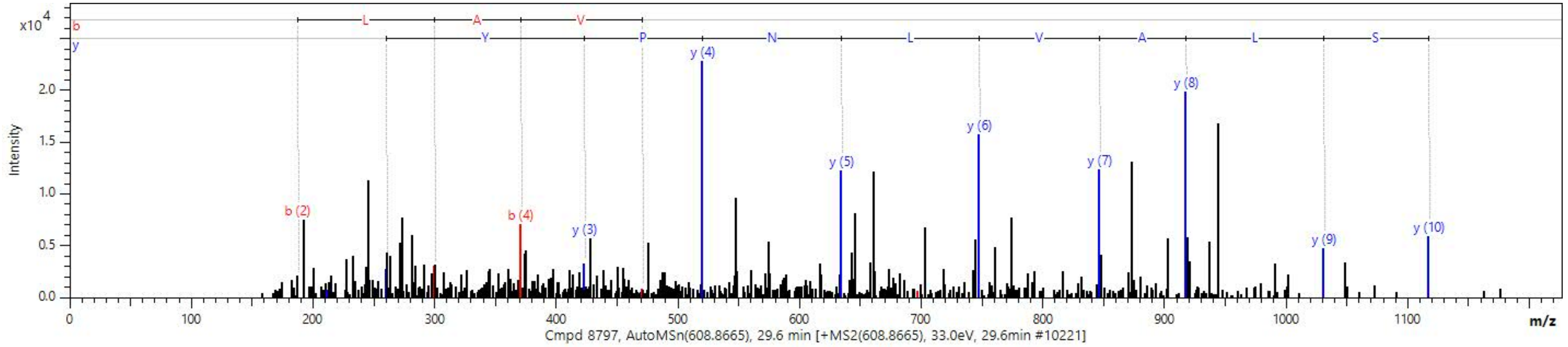

MS/MS spectra of selected mouse peptides detected in the suspension of co-cultued CCRF-CEM cells

Sequence of mouse peptide	Sequence of the closest human peptide	UniProt ID	Gene name - mouse	Protein names
ALG**GI**GPVDLLVNNAA**LVIM**QPFLEVTK	ALG**SV**GPVDLLVNNAA**VALL**QPFLEVTK	P08074	Cbr2	Carbonyl reductase [NADPH] 2

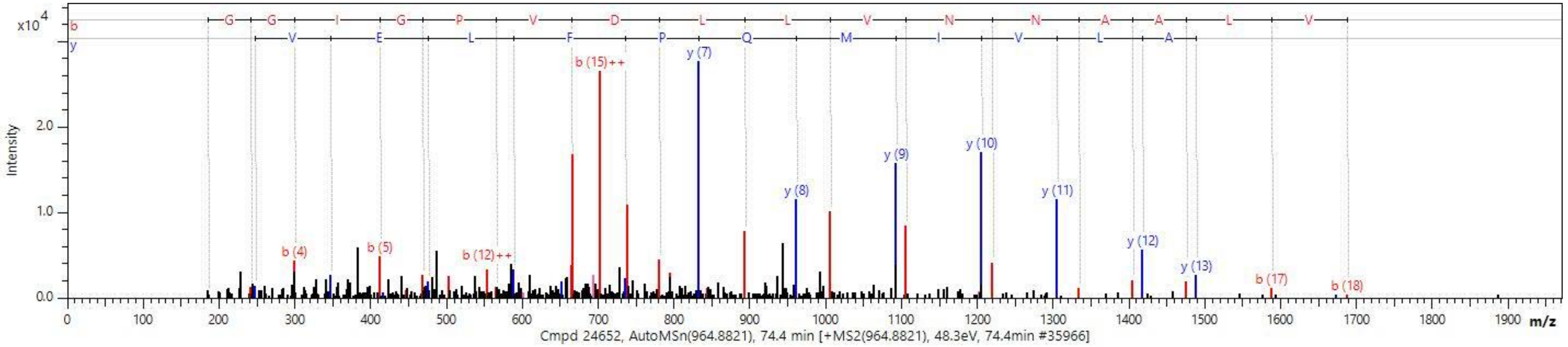

Sequence of mouse peptide	Sequence of the closest human peptide	UniProt ID	Gene name - mouse	Protein names
FAEVE**D**VVN**S**ILFLLSDR	FAEVE**H**VVN**A**ILFLLSDR	P08074	Cbr2	Carbonyl reductase [NADPH] 2

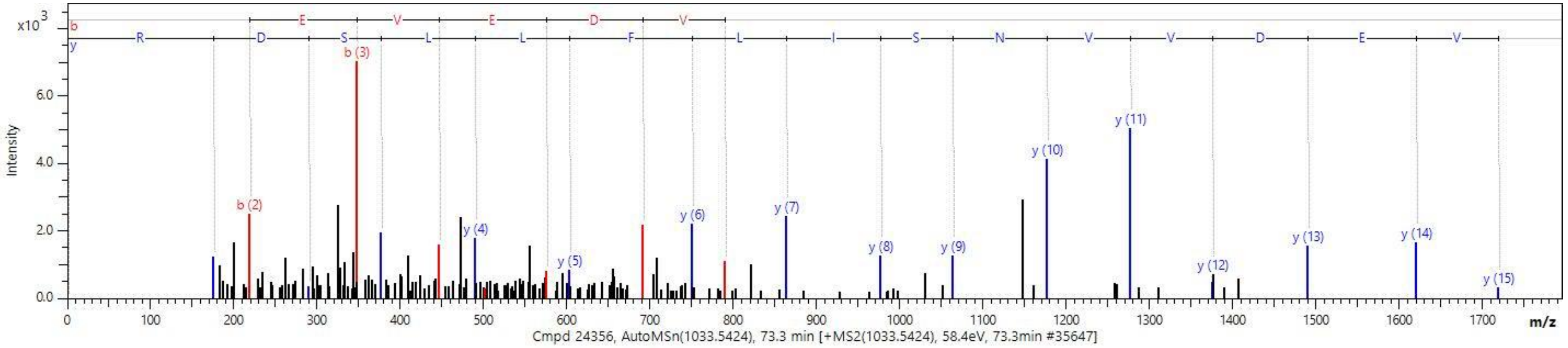

MS/MS spectra of selected mouse peptides detected in the suspension of co-cultued CCRF-CEM cells

Sequence of mouse peptide	Sequence of the closest human peptide	UniProt ID	Gene name - mouse	Protein names
TCGFDF**<u>S</u>**GA**<u>L</u>**EDISK	TCGFDF**T**GA**V**EDISK	P05202	Got2	Aspartate aminotransferase, mitochondrial

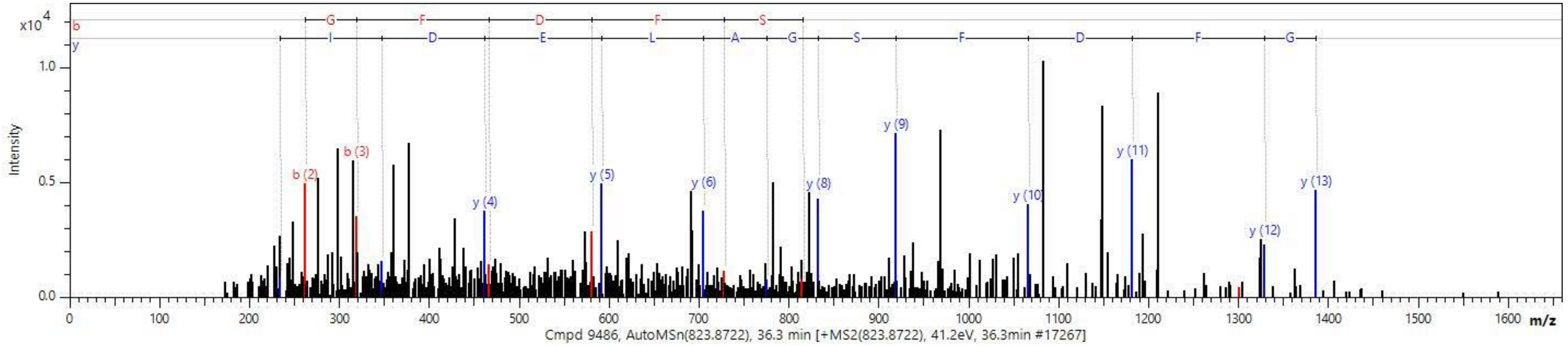

Sequence of mouse peptide	Sequence of the closest human peptide	UniProt ID	Gene name - mouse	Protein names
**E**VM**<u>Q</u>**MLV**<u>E</u>**LAK	**D**VM**R**MLV**D**LAK	P06745	Gpi	Glucose-6-phosphate isomerase

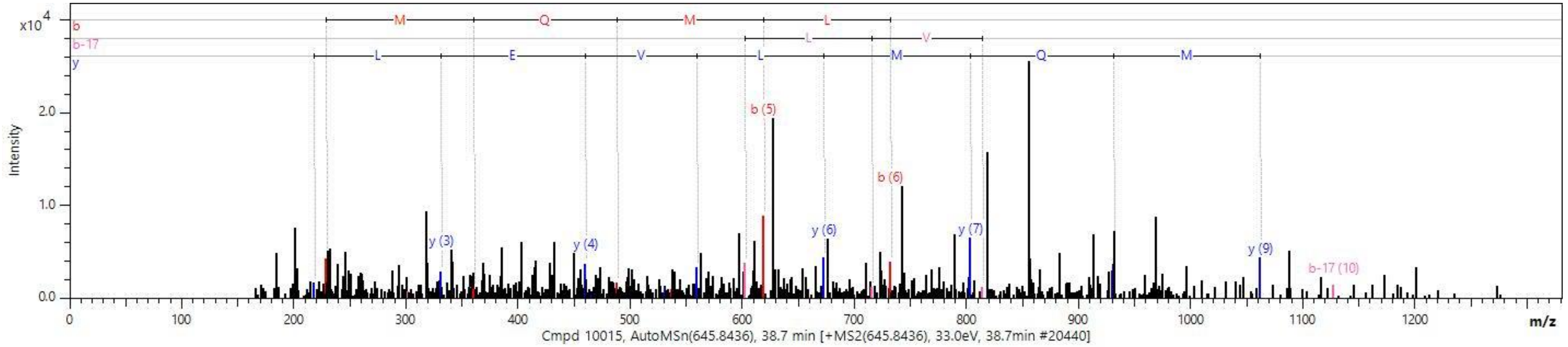

MS/MS spectra of selected mouse peptides detected in the suspension of co-cultued CCRF-CEM cells

Sequence of mouse peptide	Sequence of the closest human peptide	UniProt ID	Gene name - mouse	Protein names
LD**A**LTT**G**FGFPVGAATL**A**DEVGVDVA**Q**HVAEDLGK	LD**S**LTT**S**FGFPVGAATL**V**DEVGVDVA**K**HVAEDLGK	Q8BMS1	Hadha	Trifunctional enzyme subunit alpha, mitochondrial

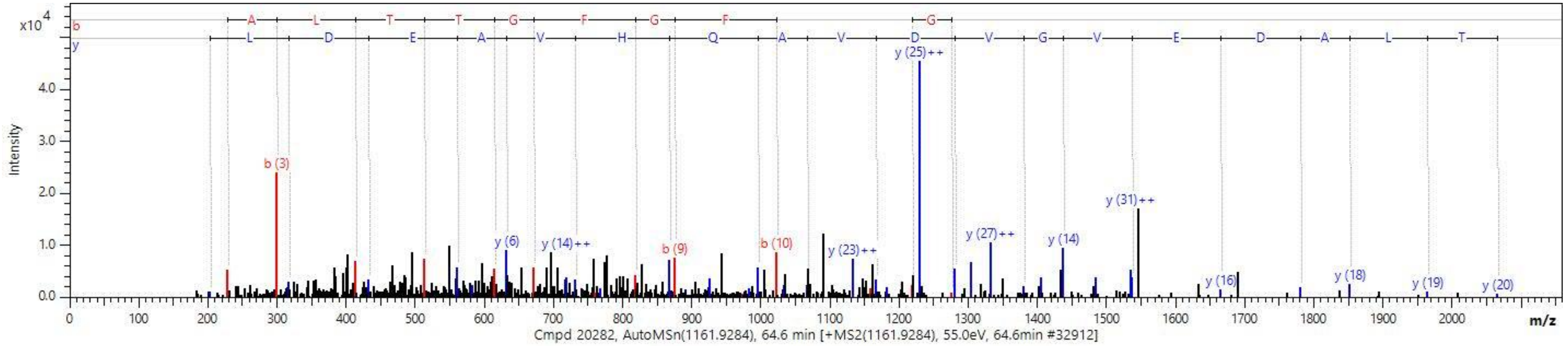

Sequence of mouse peptide	Sequence of the closest human peptide	UniProt ID	Gene name - mouse	Protein names
DLADELALVDV**M**EDK	DLADELALVDV**I**EDK	P06151	Ldha	L-lactate dehydrogenase A chain;L-lactate dehydrogenase

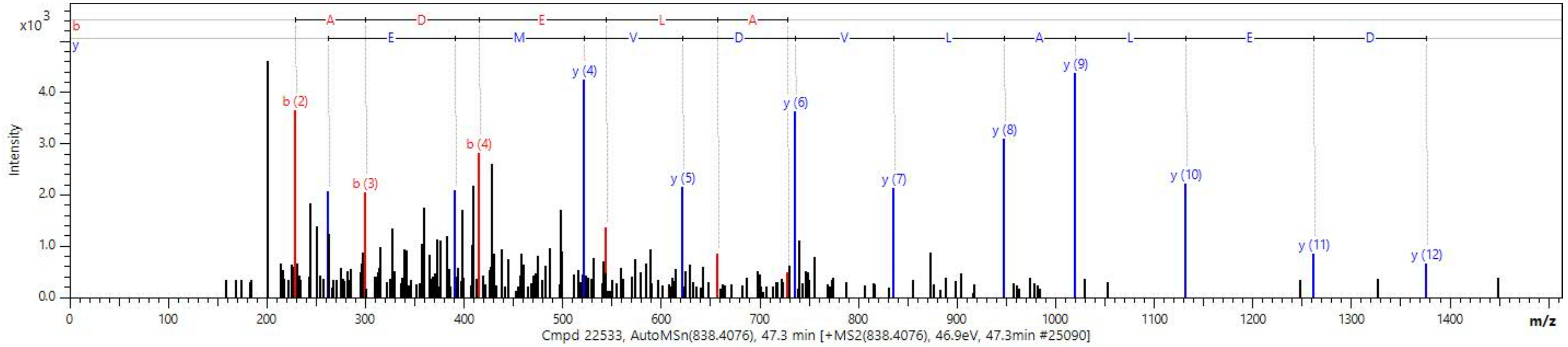

MS/MS spectra of selected mouse peptides detected in the suspension of co-cultued CCRF-CEM cells

Sequence of mouse peptide	Sequence of the closest human peptide	UniProt ID	Gene name - mouse	Protein names
DQ**L**IVNLLK	DQ**A**IVNLLK	P06151	Ldha	L-lactate dehydrogenase A chain;L-lactate dehydrogenase

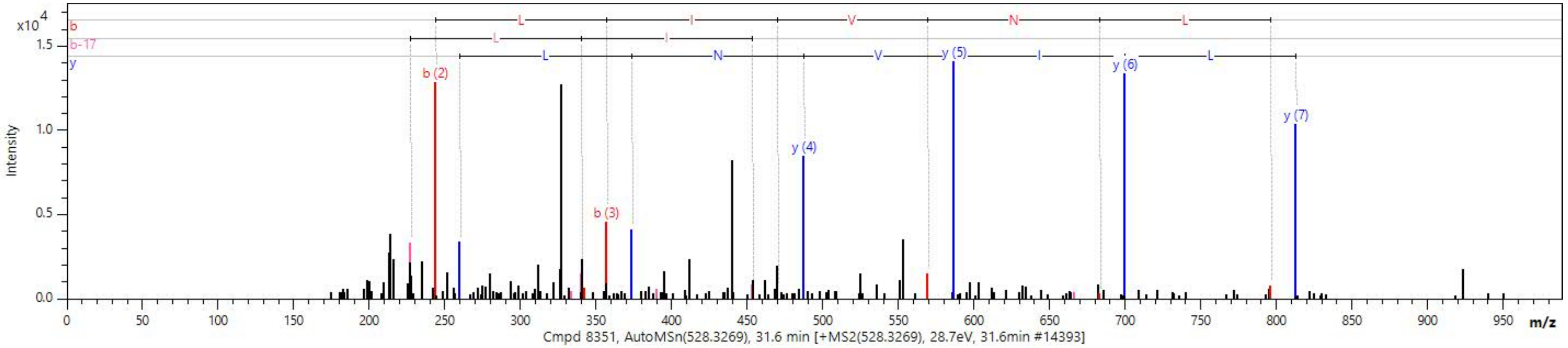

Sequence of mouse peptide	Sequence of the closest human peptide	UniProt ID	Gene name - mouse	Protein names
IQVLVE**A**DHFK	IQVLVE**P**DHFK	Q8C253	Lgals3	Galectin;Galectin-3

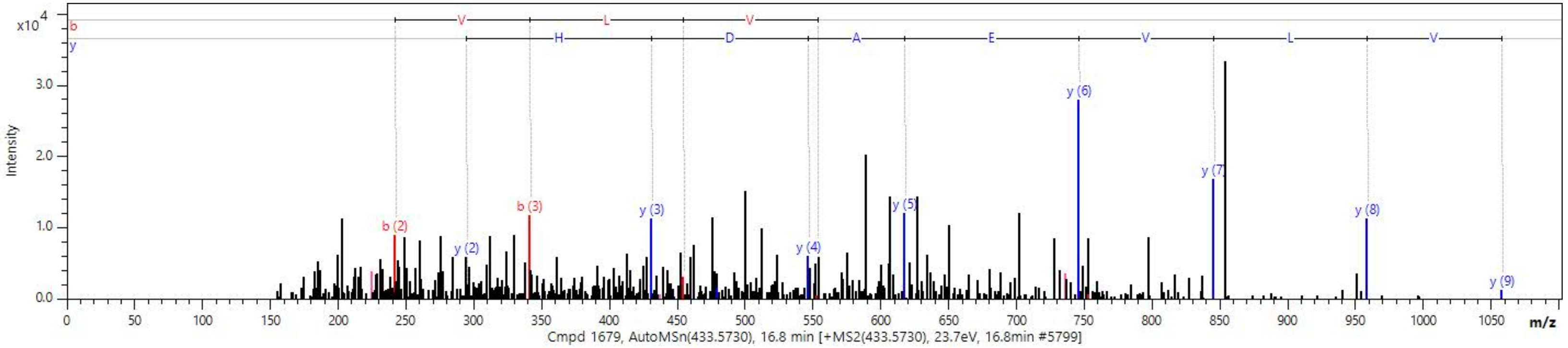

MS/MS spectra of selected mouse peptides detected in the suspension of co-cultued CCRF-CEM cells

Sequence of mouse peptide	Sequence of the closest human peptide	UniProt ID	Gene name - mouse	Protein names
QIQI**A**QLFGVPVVVALNVFK	QIQI**T**QLFGVPVVVALNVFK	Q3V3R1	Mthfd1l	Monofunctional C1-tetrahydrofolate synthase, mitochondrial

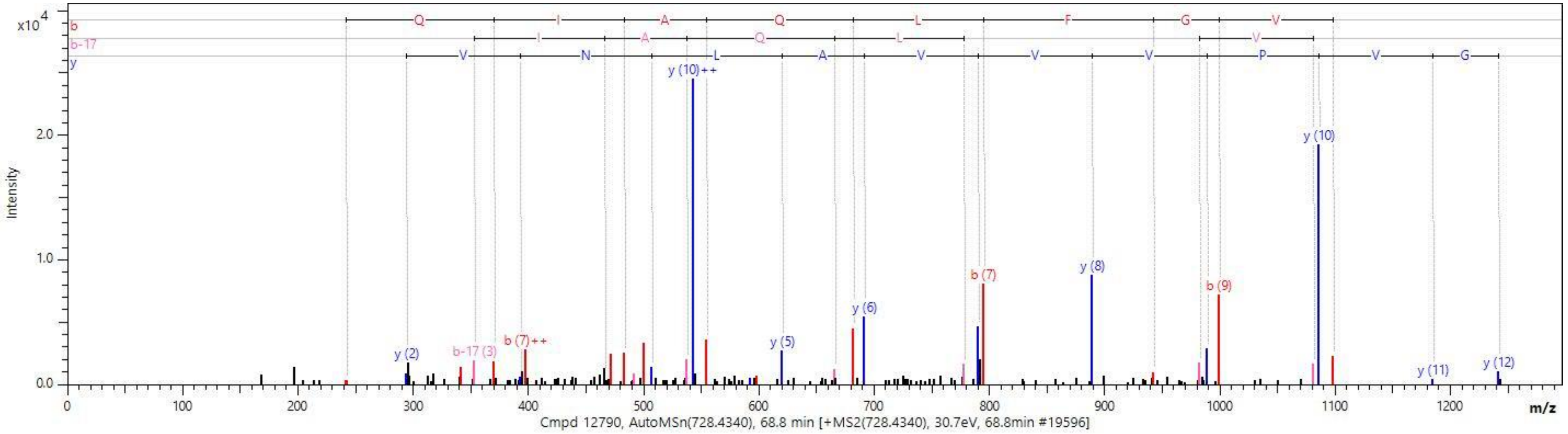

Sequence of mouse peptide	Sequence of the closest human peptide	UniProt ID	Gene name - mouse	Protein names
FAISE**L**FAK	FAI**TVSL**SE**M**FAK	P09405	Ncl	Nucleolin

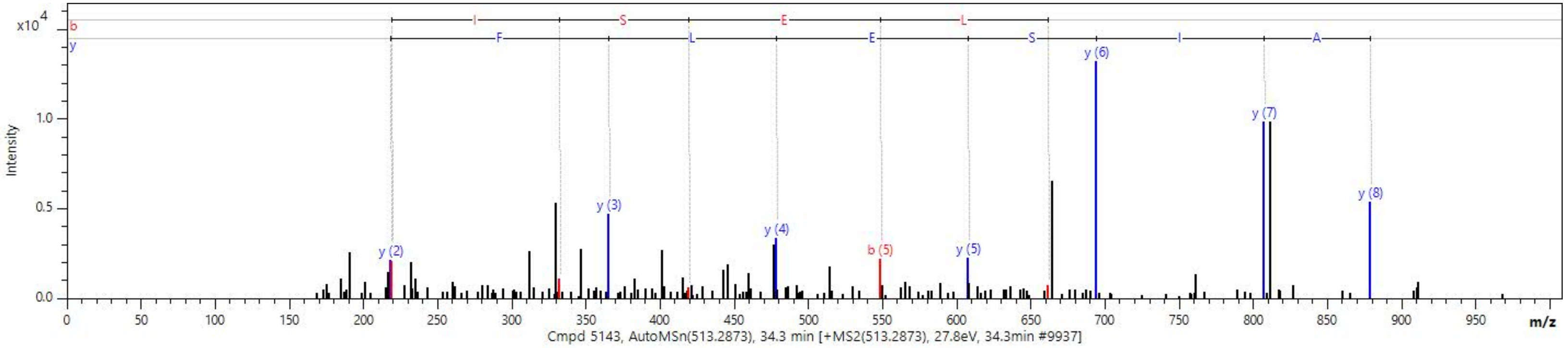

MS/MS spectra of selected mouse peptides detected in the suspension of co-cultued CCRF-CEM cells

Sequence of mouse peptide	Sequence of the closest human peptide	UniProt ID	Gene name - mouse	Protein names
QFL**L**AAEAIDDIPFGITSNS**G**VFSK	QFL**Q**AAEAIDDIPFGITSNS**D**VFSK	P09103	P4hb	Protein disulfide-isomerase

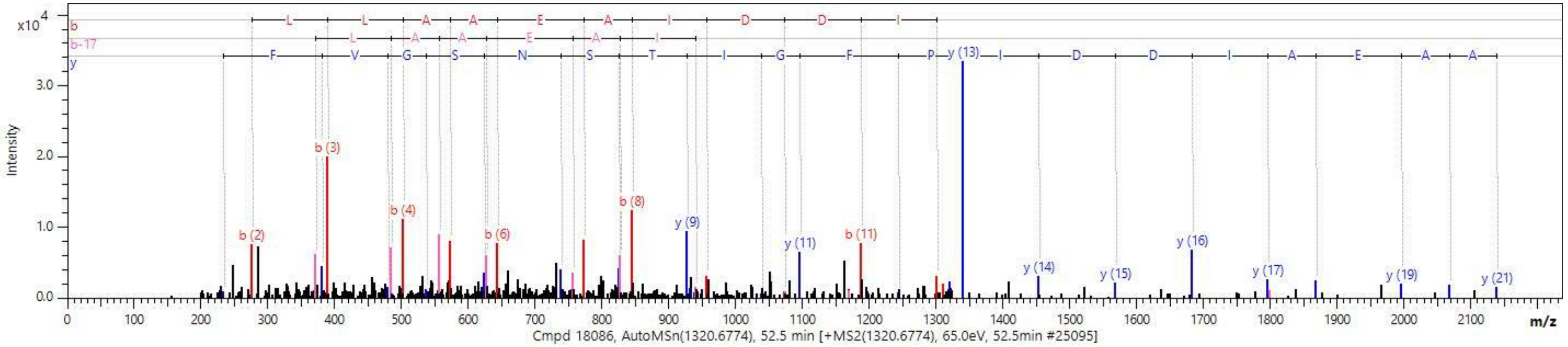

Sequence of mouse peptide	Sequence of the closest human peptide	UniProt ID	Gene name - mouse	Protein names
TGPAATTL**S**D**T**AAAESLV**D**SSEV**T**VIGFFK	TGPAATTL**P**D**G**AAAESLV**E**SSEV**A**VIGFFK	P09103	P4hb	Protein disulfide-isomerase

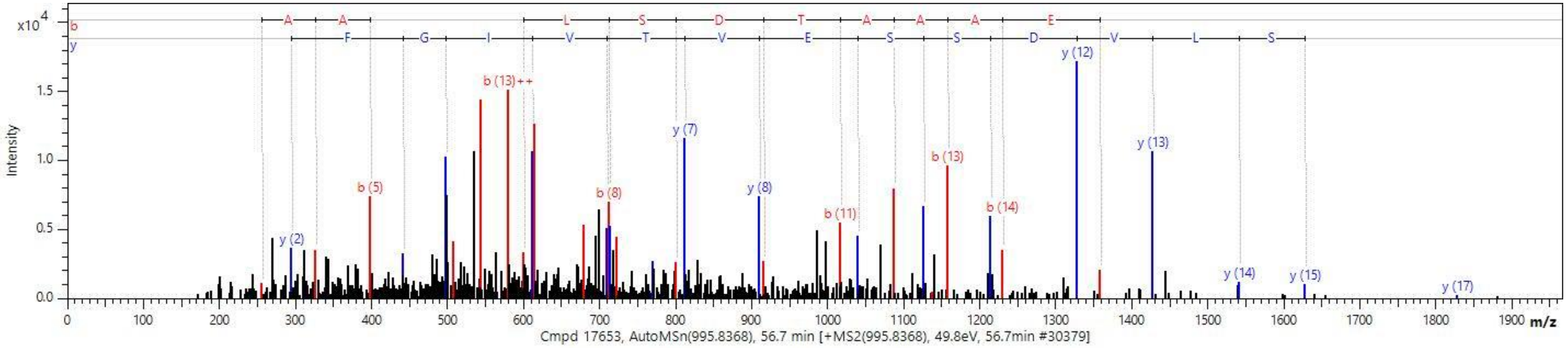

MS/MS spectra of selected mouse peptides detected in the suspension of co-cultued CCRF-CEM cells

Sequence of mouse peptide	Sequence of the closest human peptide	UniProt ID	Gene name - mouse	Protein names
ALE**<u>Q</u>**FLQ**<u>E</u>**YFDGNLK	ALE**R**FLQ**D**YFDGNLK	P27773	Pdia3	Protein disulfide-isomerase A3

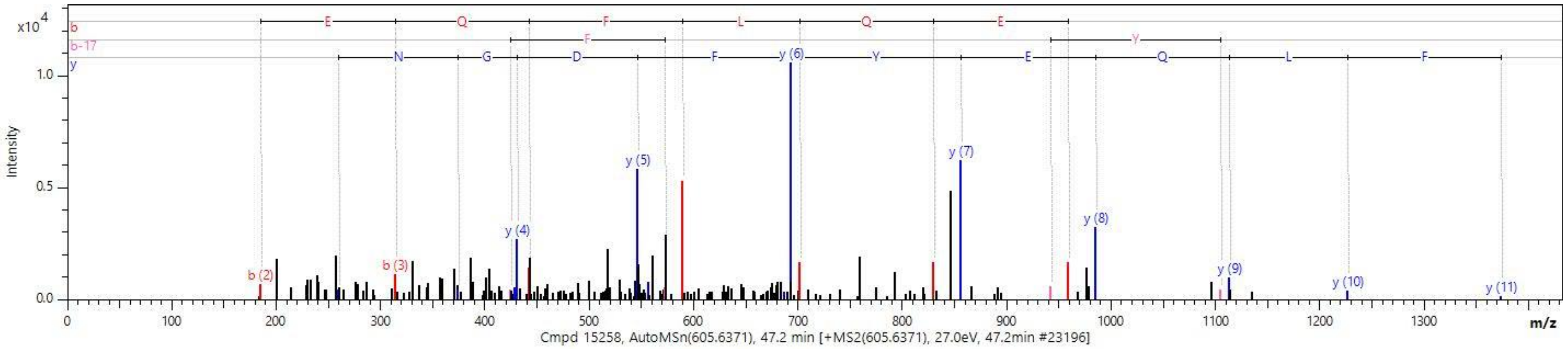

Sequence of mouse peptide	Sequence of the closest human peptide	UniProt ID	Gene name - mouse	Protein names
DGELPVEDDIDLSDVELDDL**<u>E</u>**KDEL	DGELPVEDDIDLSDVELDDL**G**KDEL	Q922R8	Pdia6	Protein disulfide-isomerase A6

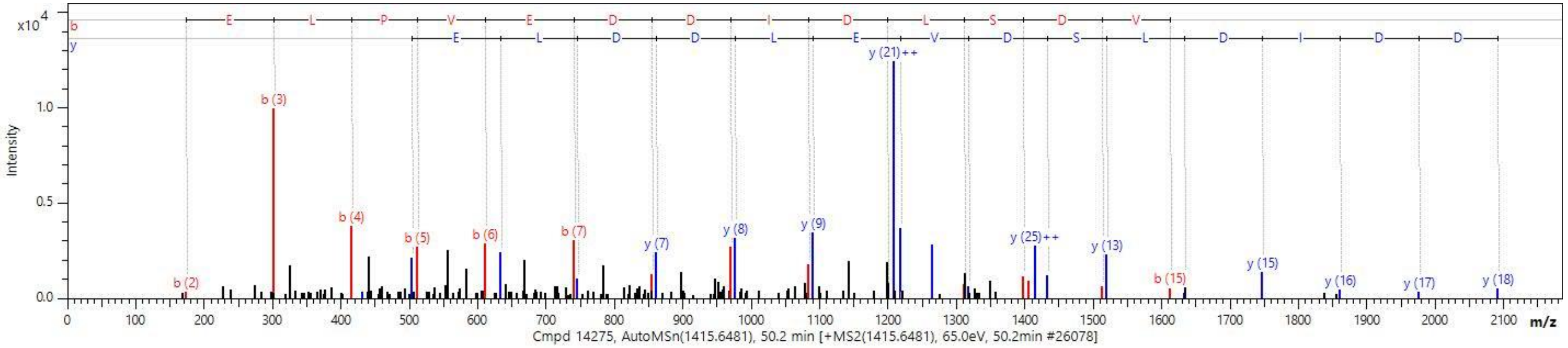

MS/MS spectra of selected mouse peptides detected in the suspension of co-cultued CCRF-CEM cells

Sequence of mouse peptide	Sequence of the closest human peptide	UniProt ID	Gene name - mouse	Protein names
TFV**S**ITPAEVGVLVGK	TFV**N**ITPAEVGVLVGK	P62962	Pfn1	Profilin-1;Profilin

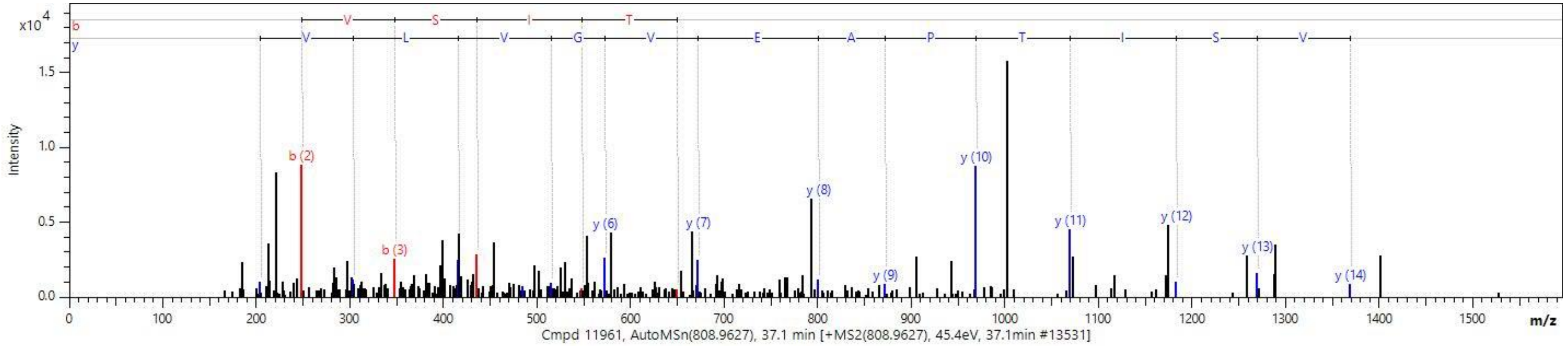

Sequence of mouse peptide	Sequence of the closest human peptide	UniProt ID	Gene name - mouse	Protein names
VTLTLPVLNAA**QSI**IFVATGEGK	VTLTLPVLNAA**RTV**IFVATGEGK	Q9CQ60	Pgls	6-phosphogluconolactonase

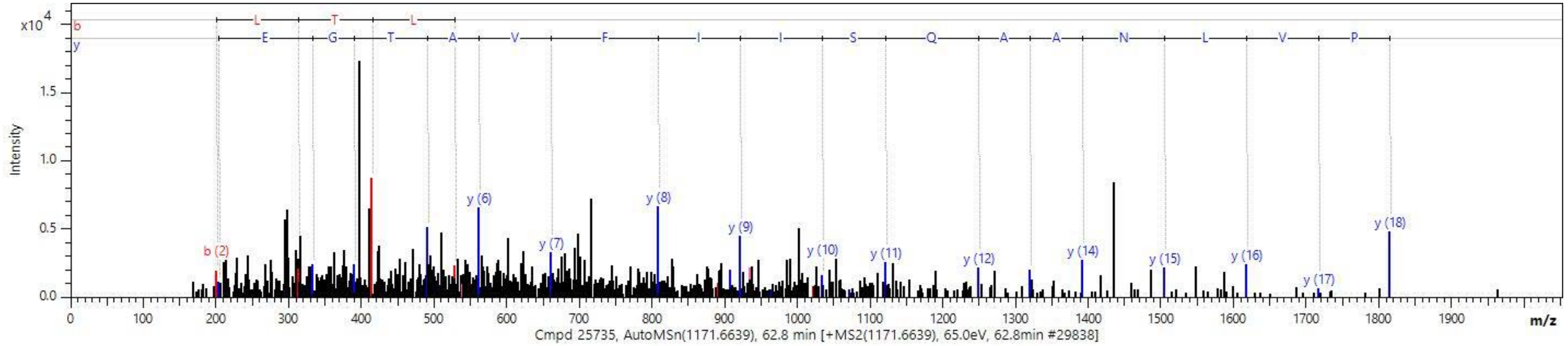

MS/MS spectra of selected mouse peptides detected in the suspension of co-cultued CCRF-CEM cells

Sequence of mouse peptide	Sequence of the closest human peptide	UniProt ID	Gene name - mouse	Protein names
ESADPL**S**AWLQDA**K**	ESADPL**G**AWLQDA**R**	Q9QXS1	Plec	Plectin

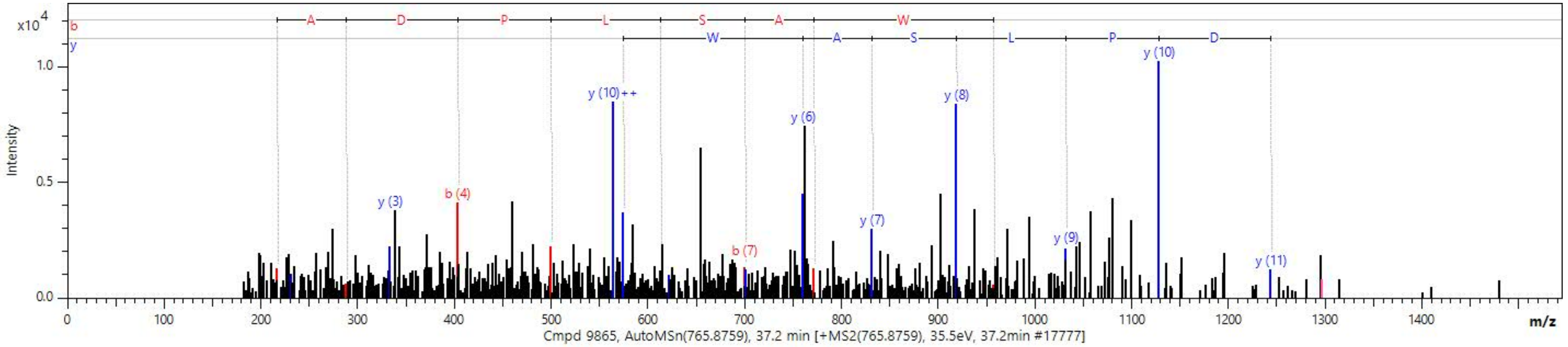

Sequence of mouse peptide	Sequence of the closest human peptide	UniProt ID	Gene name - mouse	Protein names
DQEVNFQEYV**A**FLGALALIYNEALK	DQEVNFQEYV**T**FLGALALIYNEALK	P14069	S100a6	Protein S100-A6

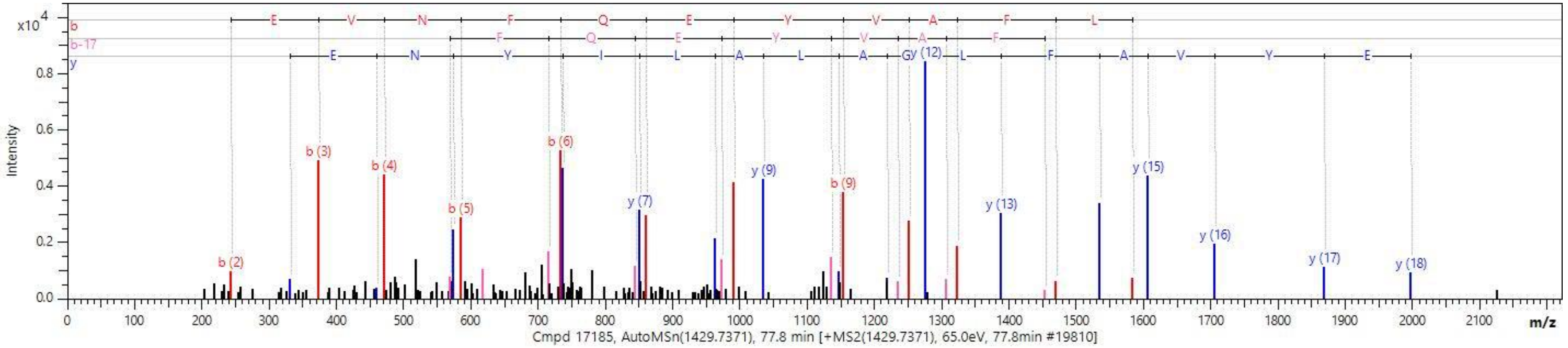

MS/MS spectra of selected mouse peptides detected in the suspension of co-cultued CCRF-CEM cells

Sequence of mouse peptide	Sequence of the closest human peptide	UniProt ID	Gene name - mouse	Protein names
LNP**Q**TGLIDY**D**QLALTAR	LNP**K**TGLIDY**N**QLALTAR	Q9CZN7	Shmt2	Serine hydroxymethyltransferase

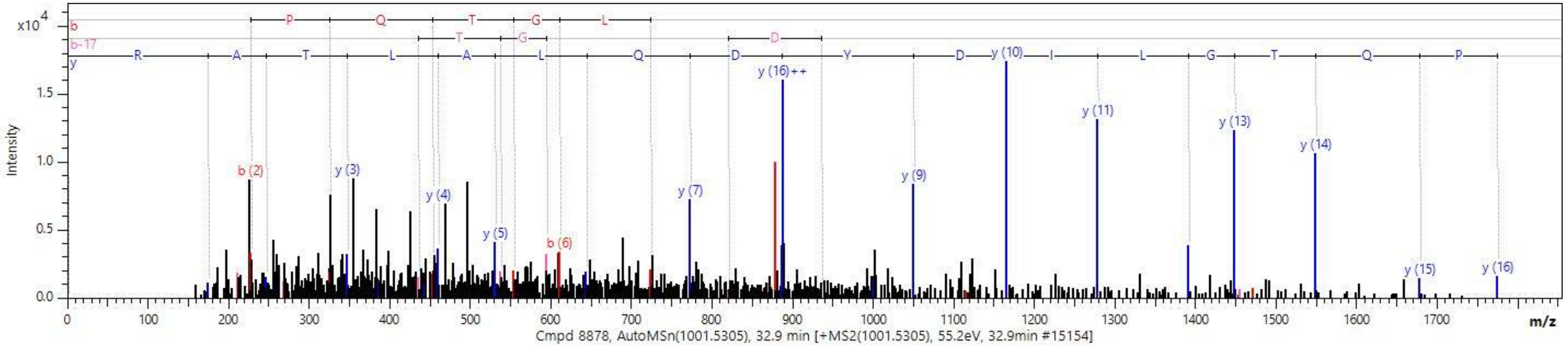

Sequence of mouse peptide	Sequence of the closest human peptide	UniProt ID	Gene name - mouse	Protein names
**C**LGELI**C**TLNAA**N**VPA**G**TEVVCAPPTAYIDFAR	**S**LGELI**G**TLNAA**K**VPA**D**TEVVCAPPTAYIDFAR	P17751	Tpi1	Triosephosphate isomerase

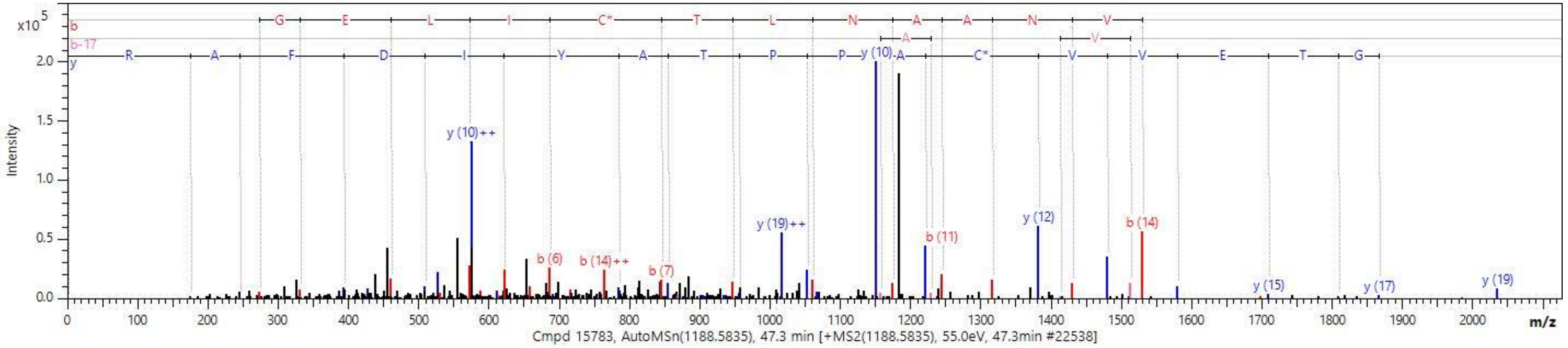

MS/MS spectra of selected mouse peptides detected in the suspension of co-cultued CCRF-CEM cells

Sequence of mouse peptide	Sequence of the closest human peptide	UniProt ID	Gene name - mouse	Protein names
AQE**Q**LATALQ**_N_**LEEAEK	AQE**R**LATALQ**K**LEEAEK	Q6IRU2	Tpm4	Tropomyosin alpha-4 chain

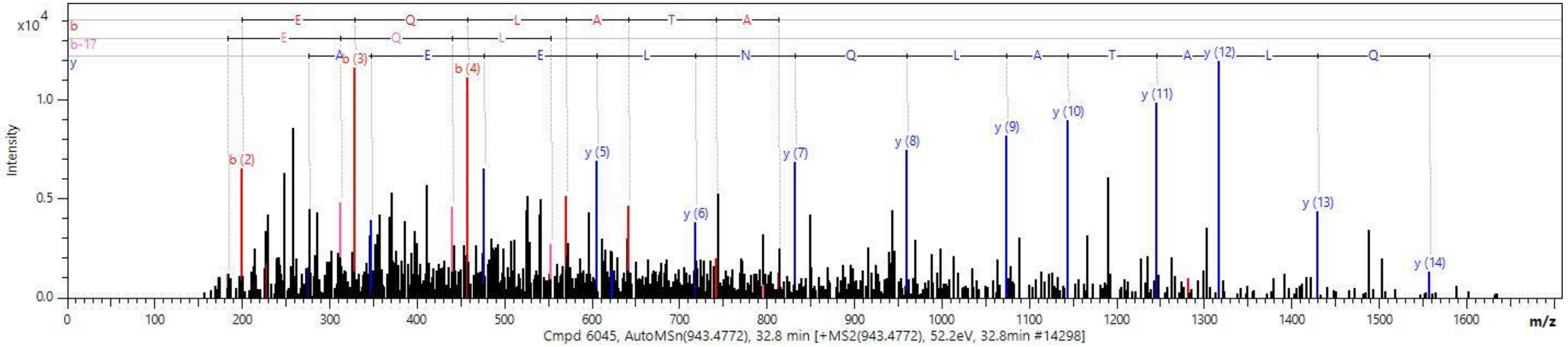

Supplement: Supplementary file 1 [file proteomes-11-00015-s001.zip › Supplementary Figure S1.pdf]
