# Supplementary figures and images for: Shotgun Proteomics of Co-Cultured Leukemic and Bone Marrow Stromal Cells from Different Species as a Preliminary Approach to Detect Intercellular Protein Transfer
Source: Proteomes. 2023 Apr 5;11(2):15. doi: 10.3390/proteomes11020015 (PMC10123657; doi:10.3390/proteomes11020015)

# Supplementary Figure S2. Uncropped images of Western blot experiments.

## A

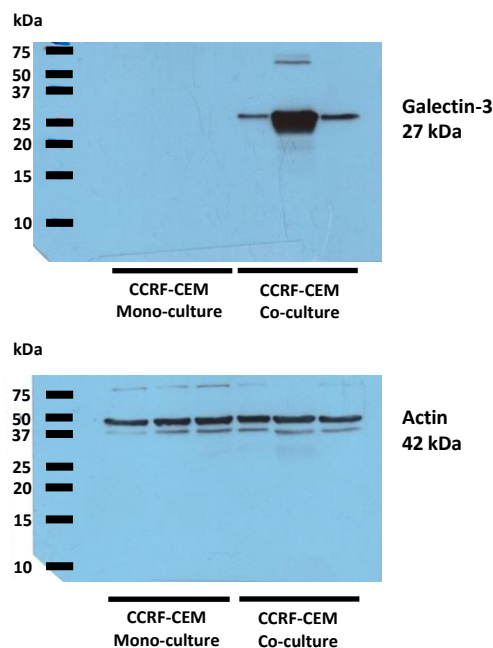

## B

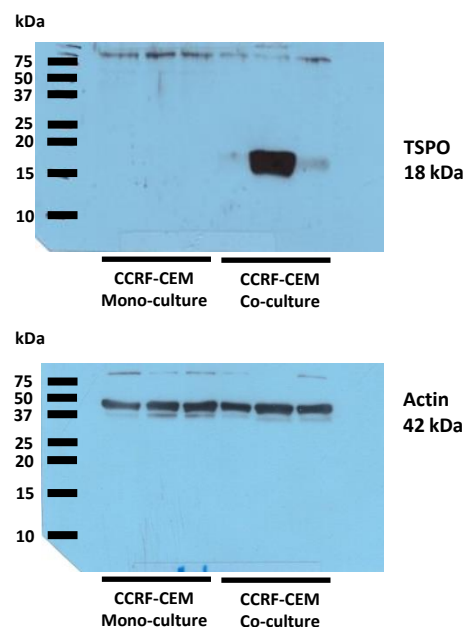

## C

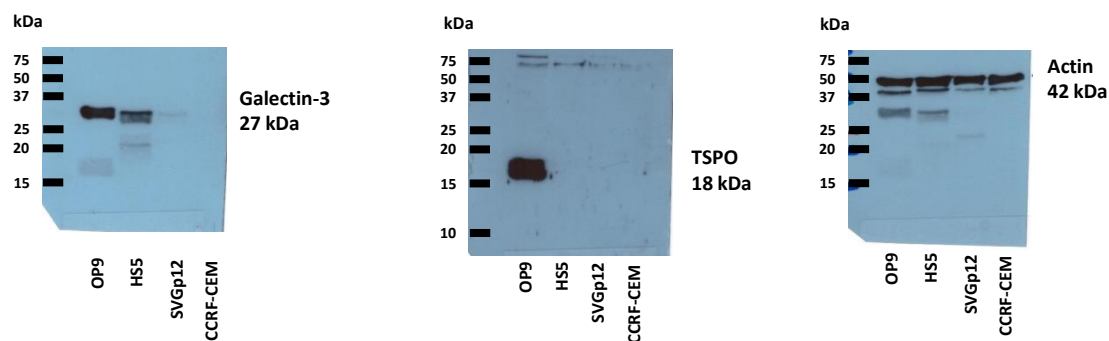

Supplement: Supplementary file 1 [file proteomes-11-00015-s001.zip › Supplementary Figure S2.pdf]
